# Supplementary material for: Origin, structure and functional transition of sex pheromone components in a false widow spider
Source: Commun Biol. 2022 Oct 30;5:1156. doi: 10.1038/s42003-022-04072-7 (PMC9618557; doi:10.1038/s42003-022-04072-7)
Supplement: Supplementary file 2 — Supplementary Materials [file 42003_2022_4072_MOESM2_ESM.pdf]

Origin, structure, and functional transition of sex pheromone components in a false widow spider

Andreas Fischer[1]*, Regine Gries[1], Santosh K. Alamsetti[1], Emmanuel Hung[1], Andrea C. R. Torres[1], Sula Fernando[1], Sanam Meraj[1], Weiwu Ren[2,3], Robert Britton[2], Gerhard Gries[1]

[1]Department of Biological Sciences, Simon Fraser University, Vancouver, BC, Canada
[2]Department of Chemistry, Simon Fraser University, Vancouver, BC, Canada
[3]New address: School of Medicine and Pharmacy, Ocean University of China, Qingdao, China
*Corresponding Author: afischer@sfu.ca

Supplementary Table 1: List of chemicals referred to in this study, with amounts (where applicable) quantified on the web of a female *Steatoda grossa.*

#	Chemical name	Amount per web	Supplier	Purity
**1**	[(*R*)-3-Hydroxybutyryloxy]-butyric acid	N/A	N/A	N/A
**2**	*N*-3-Methyl-butyryl-*O*-(*S*)-2-methylbutyryl-L-serine methyl ester	N/A	N/A	N/A
**3**	*N*-3-Methylbutanoyl-*O*-methylpropanoyl-L-serine methyl ester	N/A	N/A	N/A
**4**	(*R*)-3-Hydroxybutyric acid	N/A	N/A	N/A
**5**	Pyrrolidin-2-one	4,000 ng	Sig-Ald[1]	99%
**6**	4-Hydroxyhydrofuran-2(3*H*)-one	200 ng	Sig-Ald	95%
**7**	Nonanoic acid	20 ng	Sig-Ald	≥ 97%
**8**	Dodecanoic acid	100 ng	Sig-Ald	98%
**9**	6-Methylheptanamide	20 ng	Gries-lab	>95%
**10**	Octanamide	40 ng	Gries-lab	>95%
**11**	4,6-dimethylheptanamide	40 ng	Gries-lab	>95%
**12**	*N*-4-Methylvaleroyl-*O*-butyroyl-L-serine	145 ng	Gries-lab	90%
**13**	*N*-4-Methylvaleroyl-*O*-butyroyl-L-serine methyl ester[2]	N/A	Gries-lab	N/A
**14**	*N*-4-Methylvaleroyl-*O*-isobutyroyl-L-serine methyl ester[2]	N/A	Gries-lab	N/A
**15**	*N*-4-Methylvaleroyl-*O*-hexanoyl-L-serine methyl ester[2]	N/A	Gries-lab	N/A
**16**	*N*-4-Methylvaleroyl-*O*-isobutyroyl-L-serine	20 ng	Gries-lab	>90%
**17**	*N*-4-Methylvaleroyl-*O*-hexanoyl-L-serine	20 ng	Gries-lab	>90%
**18**	*N*-4-Methylvaleroyl-L-serine	200 ng	Gries-lab	>82
**19**	Butyric acid	103 ng	Sig-Ald	> 99%
**20**	Isobutyric acid	3 ng	Sig-Ald	99%
**21**	Hexanoic acid	54 ng	Sig-Ald	> 99%

[1]Sigma-Aldrich
[2]Prepared by diazomethane treatment of **12**, **16** and **17** (compounds were not tested in bioassays)

Supplementary Table 2 **Summary of behavioral experiments and analytical procedures.** List of compounds (bold-face) tested and materials analyzed, type of bioassay apparatus [T-rod (Fig. 1 c); Y-tube olfactometer (Fig. 4 d) and analytical instruments used for behavioral experiments and chemical analyses, respectively, and statistical procedures applied for data analyses.

**Exp. #**	**Assay/analysis**	**Compounds[1,2]/material tested**	**Statistical analyses**
Identification of contact pheromone components			
1	T-rod	**5-11**	Wilcoxon rank sum test<br>N = 20, W = 370, $P < 0.001$
2	T-rod	Web extract	
3	T-rod	Web extract	
4	T-rod	**12**, **16**, **17**	Kruskal-Wallis χ^2 test[4]
5	T-rod	**5-11** + **12**, **16**, **17**	$\chi^2 = 35.068$, df = 3, $P < 0.001$
6	T-rod	**5-11**	
7	T-rod	**12**, **16**, **17** (10 FWE)	
8	T-rod	**12**, **16**, **17** (1 FWE)	
9	T-rod	**12**, **16**, **17** (0.1 FWE)	Kruskal-Wallis χ^2 test[4]<br>$\chi^2 = 61.750$, df = 4, $P < 0.001$
10	T-rod	**12**, **16**, **17** (0.01 FWE)	
11	T-rod	**12**, **16**, **17** (0.001 FWE)	
12	T-rod	**12**, **16**, **17**	
13	T-rod	**12**, **17**	Kruskal-Wallis χ^2 test[4]
14	T-rod	**12**, **16**	$\chi^2 = 11.191$, df = 3, $P = 0.010$
15	T-rod	**16**, **17**	
16	T-rod	**12**, **16**	
17	T-rod	**12**	Kruskal-Wallis χ^2 test[4]<br>$\chi^2 = 3.652$, df = 2, $P = 0.160$
18	T-rod	**16**	
Origin of contact pheromone components			
19	HPLC-MS[3]	Spider tagmata	Wilcoxon rank sum test<br>N = 22, W = 21, $P = 0.004$
20	HPLC-MS	Abdominal tissues	Kruskal-Wallis χ^2 test[4]<br>$\chi^2 = 70.96$, df = 6, $P < 0.001$
21	HPLC-MS	Silk glands	Kruskal-Wallis χ^2 test[4]<br>$\chi^2 = 36.00$, df = 6, $P < 0.001$
Transition of contact pheromone components to mate attractant pheromone components			
22	Y-tube	Web extract	One-sided binomial test: $P = 0.013$

23	Y-tube	**5-11**	One-sided binomial test: P = 0.588
24	HPLC-MS	**18** / (**18**+**12**+**16**+**17**)	Wilcoxon rank sum test W= 638, N = 70, $P < 0.001$
25	Y-tube	**18-21**	One-sided binomial test: P = 0.030
26	Y-tube	**19-21**	One-sided binomial test: P = 0.006
27	Y-tube	**18**	One-sided binomial test: P = 0.500
28	Adhesive traps in hallways	**19-21**	One-sided binomial test: P = 0.011
Mechanisms underlying the transition of contact pheromone components to sex attractant pheromone components			
29	T-rod	**12**, **16**, **17**, **18**	
30	T-rod	**12**, **16**, **17**	Kruskal-Wallis χ^2 test[4] $\chi^2 = 12.78$, df = 2, $P < 0.001$
31	T-rod	**18**	
32	pH / HPLC-MS	webs/web extracts	Generalized linear model $F_{1,69} = 108.44$, $P < 0.001$
33	HPLC-MS	**12** (in pH 7 buffer solution)	
34	HPLC-MS	**12** (in pH 4 buffer solution)	Kruskal-Wallis χ^2 test[4] $\chi^2 = 25.84$, df = 2, $P < 0.001$
35	HPLC-MS	**12** (in acetonitrile)	

[1]Numbers refer to chemicals listed in STable 1
[2]Female web equivalent: amount of analyte present in the extract of a web from a single female *S. grossa*
[3]HPLC-MS: High performance liquid chromatography – mass spectrometry
[4]p-value corrected for multiple comparison using the Bonferroni-Hochberg method.

Supplementary Figures

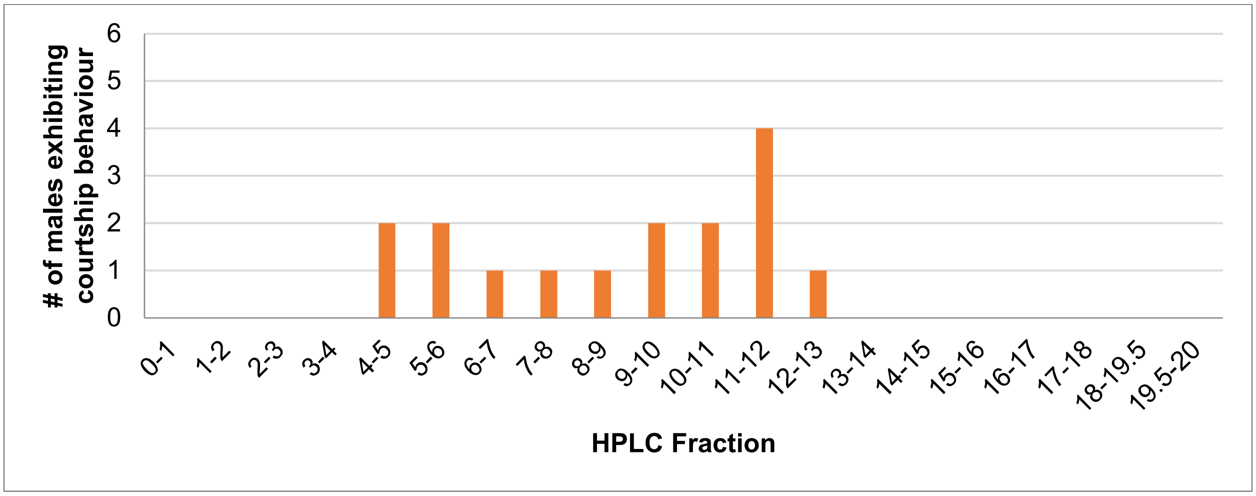

Supplementary Figure 1. **Courtship by *Steatoda grossa* males in response to HPLC fractions of female *S. grossa* web extract.** Number of males exhibiting courtship behaviour in response to high performance liquid chromatography (HPLC) fractions of crude extract of female *S. grossa* webs.

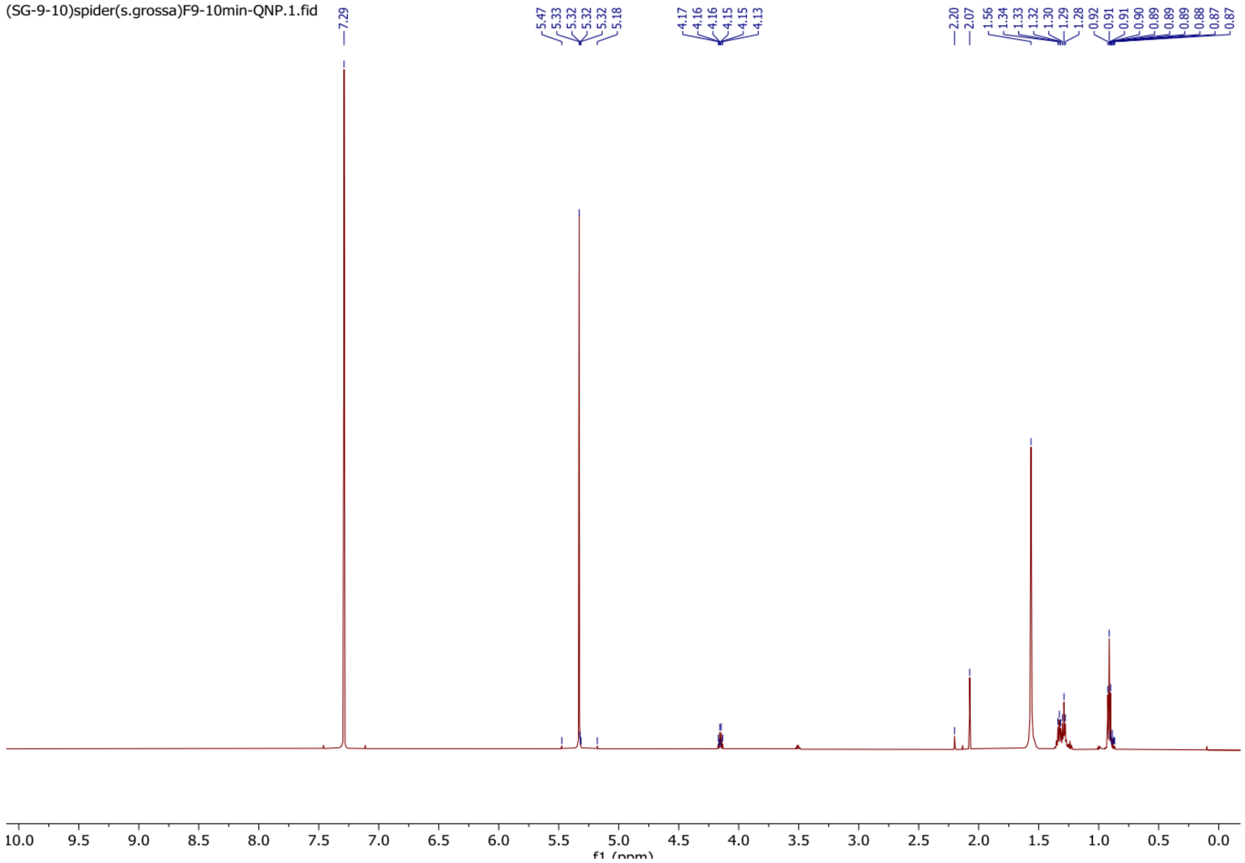

Supplementary Figure 2. **^{1}H NMR spectrum of *N*-4-methylpentanoyl-*O*-butyryl-L-serine produced by female *Steadoda grossa.*** The compound was extracted from webs of females, isolated by high performance liquid chromatography (see SFigure 1), and the ^{1}H NMR spectrum was recorded on a Bruker Avance 600 equipped with a QNP (600 MHz) using $CDCl_3$.

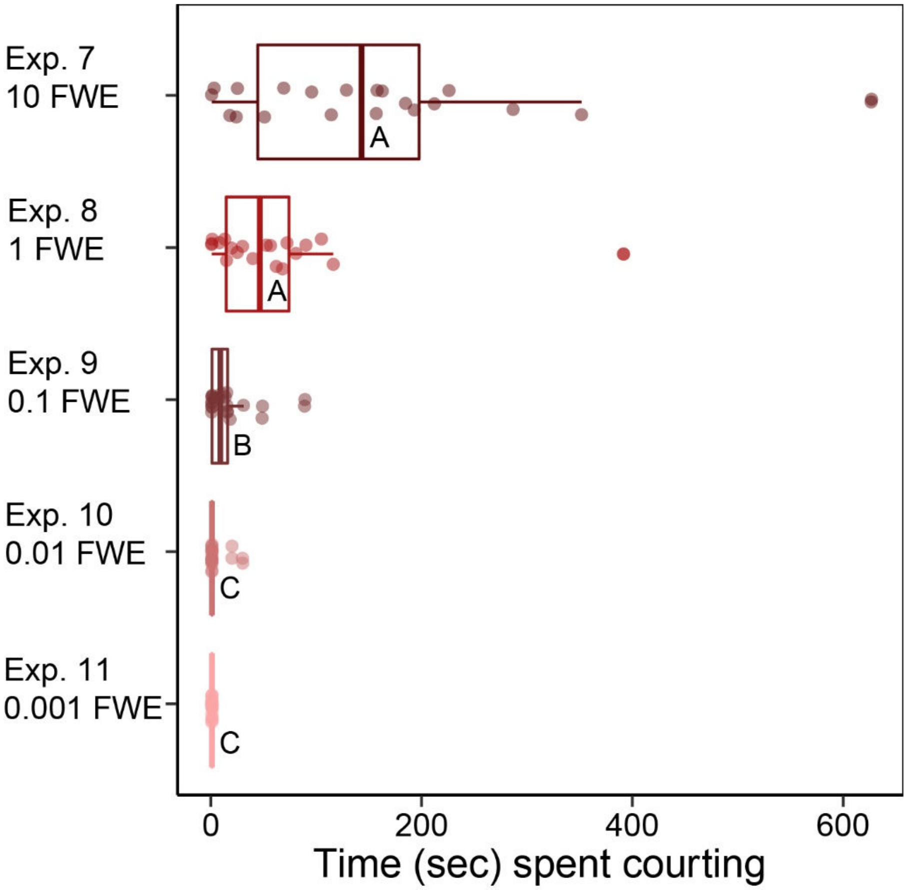

Supplementary Figure 3. **Effect of contact pheromone dose on the extent of courtship by *Steatoda grossa* males.** Time spent courting by *S. grossa* males in response to a ternary blend of synthetic contact pheromone components [*N*-4-methylvaleroyl-*O*-isobutyroyl-L-serine (**12**); *N*-4-methylvaleroyl-*O*-isobutyroyl-L-serine (**16**); *N*-4-methylvaleroyl-*O*-hexanoyl-L-serine (**17**)] tested at five levels of female web equivalents (FWEs = amount of analyte present in the extract of a web from a single female *S. grossa*). Circles and boxplots show the time single male spiders courted in each replicate and the distribution of data (minimum, first quartile, median, third quartile, maximum), respectively. Medians with different letters indicate statistically significant differences in courtship responses; Kruskal-Wallis χ^2 test with Benjamini-Hochberg correction to account for multiple comparisons.

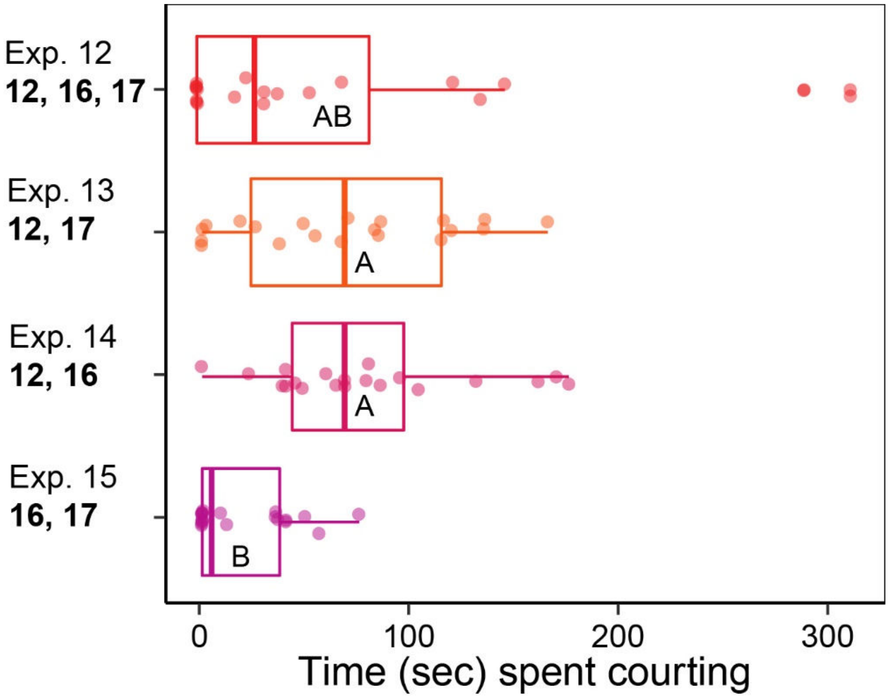

Supplementary Figure 4. **Effect of blend composition of contact pheromone components on the extent of courtship by *Steatoda grossa* males.** Time spent courting by *S. grossa* males in response to ternary and binary blends of synthetic contact pheromone components [*N*-4-methylvaleroyl-*O*-isobutyroyl-L-serine (**12**); *N*-4-methylvaleroyl-*O*-isobutyroyl-L-serine (**16**); *N*-4-methylvaleroyl-*O*-hexanoyl-L-serine (**17**)] tested at one female web equivalents (amount of analyte present in the extract of a web from a single female *S. grossa*). Circles and boxplots show the time single male spiders courted in each replicate and the distribution of data (minimum, first quartile, median, third quartile, maximum), , respectively. Medians with different letters indicate statistically significant differences in courtship responses; Kruskal-Wallis χ^2 test with Benjamini-Hochberg correction to account for multiple comparisons.

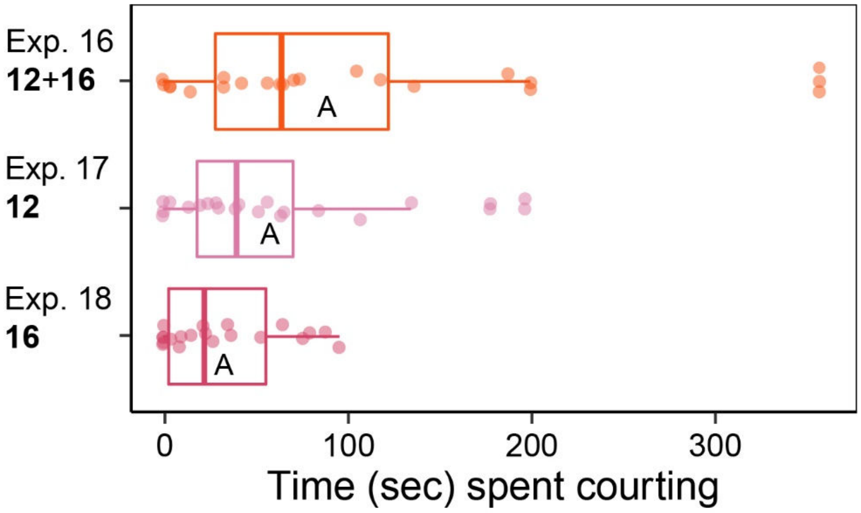

Supplementary Figure 5. **Effect of contact pheromone component(s) on the extent of courtship by *Steatoda grossa* males.** Time spent courting by *S. grossa* males in response to synthetic contact pheromone components [*N*-4-methylvaleroyl-*O*-isobutyroyl-L-serine (**12**); *N*-4-methylvaleroyl-*O*-isobutyroyl-L-serine (**16**)] tested singly or in binary combination at one female web equivalent (amount of analyte present in the extract of a web from a single female *S. grossa*). Circles and boxplots show the time single male spiders courted in each replicate and the distribution of data (minimum, first quartile, median, third quartile, maximum), respectively. Medians with different letters indicate statistically significant differences in courtship responses; Kruskal-Wallis χ^2 test with Benjamini-Hochberg correction to account for multiple comparisons.

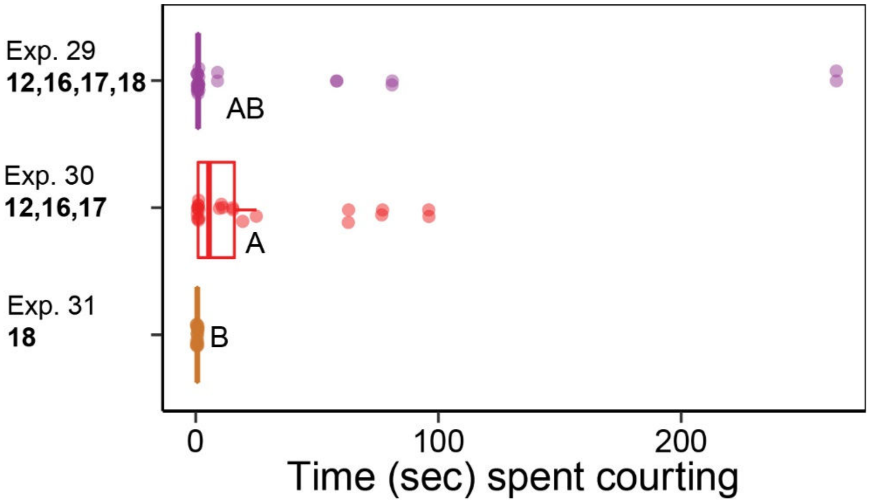

Supplementary Figure 6: **Effect of contact pheromone components and their breakdown product on the extent of courtship by *Steatoda grossa* males.** Time spent courting by *S. grossa* males in response to (i) the synthetic contact pheromone components [*N*-4-methylvaleroyl-*O*-isobutyroyl-L-serine (**12**); *N*-4-methylvaleroyl-*O*-isobutyroyl-L-serine (**16**); *N*-4-methylvaleroyl-*O*-hexanoyl-L-serine (**17**)], (ii) their breakdown product *N*-4-methylvaleroyl-L-serine (**18**) and (iii) all combined (**12**, **16**, **17**, **18**), all stimuli tested at one female web equivalents (amount of analyte present in the extract of a web from a single female *S. grossa*). Circles and boxplots show the time single male spiders courted in each replicate and the distribution of data (minimum, first quartile, median, third quartile, maximum), respectively. Means with different letters indicate statistically significant differences in courtship responses; Kruskal-Wallis χ^2 test with Benjamini-Hochberg correction to account for multiple comparisons.

Supplementary Methods: Syntheses

N-Boc-O-(*S*)-butyryl-L-serine benzyl ester

Butyric acid (3.38 mmol, 1 eq., 0.298 g) was added to a stirred mixture of N-boc-L-serine benzyl ester (3.38 mmol, 1 eq., 1.0 g) in dichloromethane (30 mL). After adding N,N'-dicyclohexylcarbodiimide (3.38 mmol, 1 eq., 0.696 g), followed by a catalytic amount of 4-(N,N-dimethylamino)pyridine, the reaction mixture was stirred 12 h at ambient temperature. Then it was purified by column chromatography with pentane/diethyl ether (2:1) as the eluent to give pure N-boc-O-(*S*)-butyryl-L-serine benzyl ester (1.1 g, 89 %). ^{1}H-NMR ($CDCl_3$, 500 MHz): δ [ppm] = 7.36-7.30 (m, 5H), 5.33 (d, J = 8.5 Hz, 1H), 5.21 (d, J = 12.2 Hz, 1H), 5.13 (d, J = 12.2

Hz, 1H), 4.60 (dt, J = 8.2, 3.8 Hz, 1H), 4.47 (dd, J = 11.2, 4.0, 1H), 4.30 (dd, J = 11.2, 3.5, 1H), 2.17 (td, J = 7.4, 5.4, 2H), 1.55 (q, J = 7.5 Hz, 2H), 1.43 (s, 9H), 0.89 (t, J = 7.4 Hz, 3H). ^{13}C NMR (126 MHz, $CDCl_3$): δ 173.0, 169.7, 155.1, 135.1, 128.6, 128.5, 128.4, 80.3, 67.5, 64.1, 53.1, 35.7, 28.3, 18.2, 13.6.

O-(*S*)-Butyryl-L-serine benzyl ester

Trifluoroacetic acid (10 mL) was added dropwise to a stirred solution of N-boc-O-(*S*)-butyryl-L-serine benzyl ester (1.0 g, 2.74 mmol) in dichloromethane (25 mL) at room temperature. The mixture was stirred 1.5 h, followed by *in vacuo* evaporation of the solvent and volatile constituents. The crude amino ester was characterized by NMR spectroscopy and used directly in the next step without purification. ^{1}H-NMR ($CDCl_3$, 500 MHz): δ [ppm] = 7.37-7.30 (m, 5H), 5.26 (d, J = 12.0 Hz, 1H), 5.19 (d, J = 12.0 Hz, 1H), 4.56 (d, J = 3.6 Hz, 2H), 4.36 (t, J = 3.6, 1H), 2.25-2.12 (m, 2H), 1.53 (h, J = 7.4 Hz, 2H), 0.87 (t, J = 7.4 Hz, 3H). ^{13}C NMR (126 MHz, $CDCl_3$): δ 173.3, 166.8, 134.0, 129.0, 128.7, 128.6, 68.9, 61.1, 52.8, 35.2, 17.9, 13.3.

N-4-Methylpentyl-O-(*S*)-butyryl-L-serine benzyl ester

Triethylamine (7.92 mmol, 3.0 eq., 0.8 g) and 4-methylpentanoyl chloride (5.28 mmol, 2.0 eq. 0.71 g) were added dropwise under stirring and ice cooling to a solution of O-(*S*)-butyryl-L-serine benzyl ester (0.7 g, 2.64 mmol) in dichloromethane (20 mL). After stirring the resulting solution 2.5 h at room temperature, the mixture was washed with a saturated solution of $NaHCO_3$ and brine, dried over $MgSO_4$, and filtered. The final product was purified by column chromatography with a mixture of pentane and diethyl ether (1:2) to yield pure N-4-methylpentyl-O-(*S*)-butyryl-L-serine benzyl ester (0.57 g, 60%). ^{1}H-NMR ($CDCl_3$, 500 MHz): δ [ppm] = 7.37-7.29 (m, 5H), 6.30 (d, J = 7.8 Hz, 1H), 5.17 (q, J = 12.0 HZ, 2H), 4.90 (dt, J = 7.6, 3.6 HZ, 1H), 4.48 (dd, J = 11.4, 4.0 Hz, 1H), 4.33 (dd, J = 11.4, 3.4, 1H), 2.26-2.21 (m, 2H), 2.16 (td, J = 7.0, 2.8 2H), 1.60-1.48 (m, 5H), 0.91-0.86 (m, 9H). ^{13}C NMR (126 MHz, $CDCl_3$) δ 173.1, 169.6, 135.0, 128.6, 128.6, 128.4, 67.6, 63.8, 51.8, 35.7, 34.4, 34.3, 27.7, 22.3, 22.3 18.2, 13.6.

N-4-Methylpentyl-O-(*S*)-butyryl-L-serine

To a solution of N-4-methylpentyl-O-(*S*)-butyryl-L-serine benzyl ester (0.5 g, 1.37 mmol) in absolute ethanol (25 mL) was added 10% Pd-C catalyst (100 mg). After stirring the reaction mixture under H_2 at room temperature overnight, the catalyst was removed by filtration over celite. Concentration under reduced pressure gave N-4-methylpentyl-O-(*S*)-butyryl-L-serine (0.33 g, 90%). ^{1}H-NMR ($CDCl_3$, 500 MHz): δ [ppm] = 8.42 (br, 2H),6.44 (d, J = 7.4 Hz, 1H), 4.85 (m, 1H), 4.52 (dd, J = 11.6, 4.4 Hz, 1H), 4.42 (dd, J = 11.6, 3.4, 1H), 2.29 (dt, J = 16.4, 7.6 4H), 1.69-1.50 (m, 3H), 0.94 (t, J = 7.4 Hz, 3H), 0.90 (d, J = 6.4 Hz, 6H). ^{13}C NMR (101 MHz, CDCl3) δ 174.5, 173.5, 171.6, 63.6, 51.9, 35.8, 34.4, 34.3, 27.7, 22.2, 22.9, 18.3, 13.5.

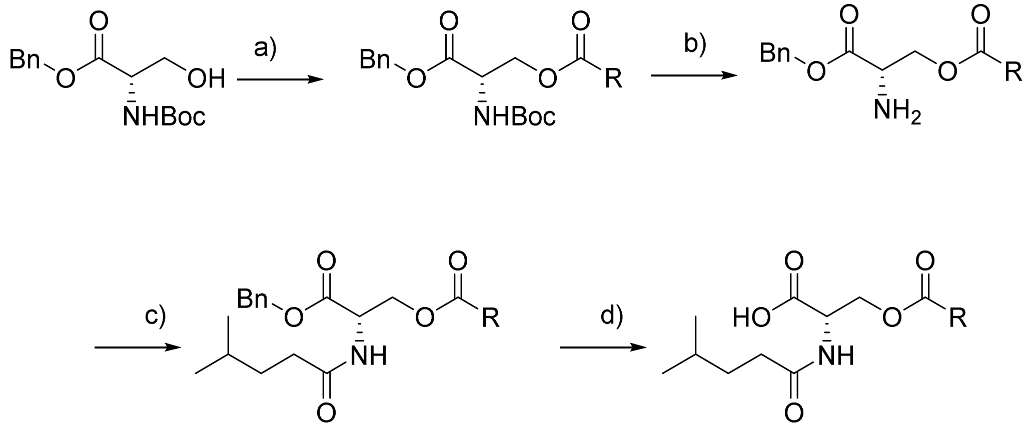

R = butyl, isobutyl or hexyl; a) butyric acid, isobutyric acid or hexanoic acid, N,N'-dicyclohexylcarbodiimide, 4-dimethylaminopyridine; b) trifluoroacetic acid; c) 4-methylpentanoyl chloride, triethylamine; d) Pd/C, hydrogen gas.

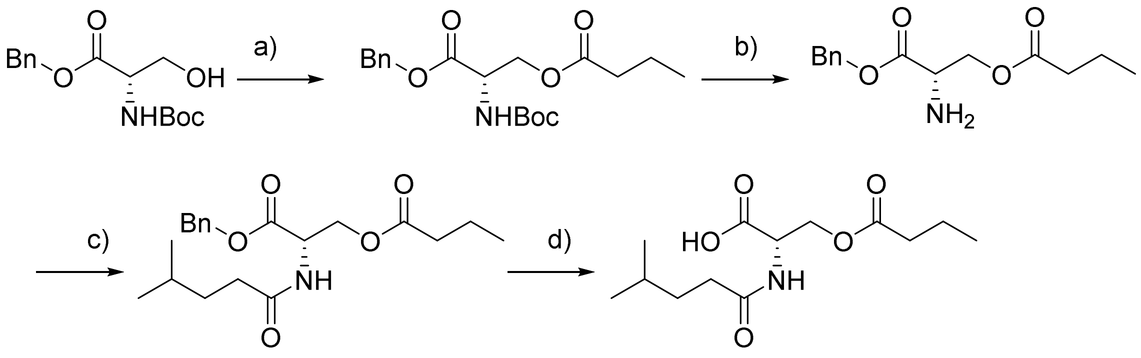

a) butyric acid, N,N'-dicyclohexylcarbodiimide,4-dimethylaminopyridine; b) trifluoroacetic acid; c) 4-methylpentanoyl chloride, triethylamine; d) Pd/C, hydrogen gas.

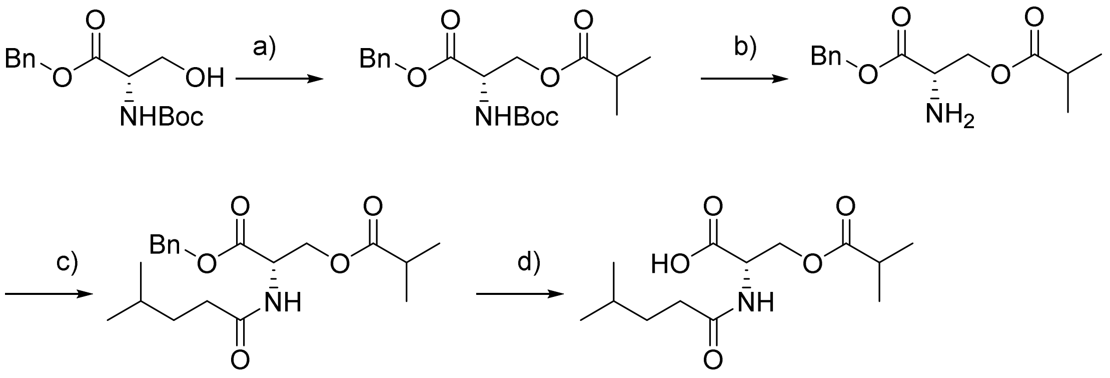

a) isobutyric acid, N,N'-dicyclohexylcarbodiimide,4-dimethylaminopyridine; b) trifluoroacetic acid; c) 4-methylpentanoyl chloride, triethylamine; d) Pd/C, hydrogen gas.

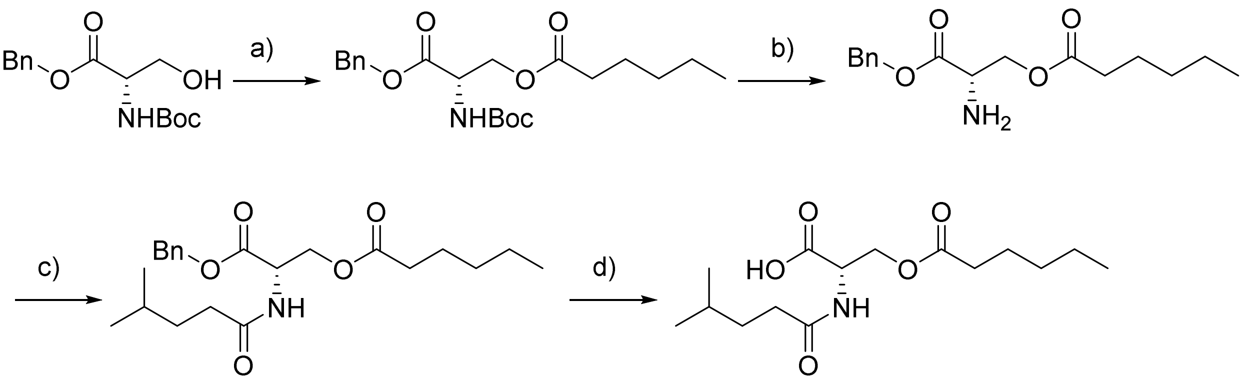

a) hexanoic acid, N,N'-dicyclohexylcarbodiimide,4-dimethylaminopyridine; b) trifluoroacetic acid; c) 4-methylpentanoyl chloride, triethylamine; d) Pd/C, hydrogen gas.

N-Boc-O-(*S*)-isobutyryl-L-serine benzyl ester

^{1}H-NMR ($CDCl_3$, 500 MHz): δ [ppm] = 7.36-7.30 (m, 5H), 5.34 (d, J = 8.5 Hz, 1H), 5.20 (d, J = 12.2 Hz, 1H), 5.13 (d, J = 12.2 Hz, 1H), 4.60 (dt, J = 8.4, 4.0 Hz, 1H), 4.46 (dd, J = 11.2, 4.0,

1H), 4.29 (dd, J = 11.2, 3.5, 1H), 2.45 (p, J = 7.0, 1H), 1.43 (s, 9H), 1.17 (d, J = 7.0 Hz, H), 1.07 (dd, J = 10.4, 7.0 Hz, 6H). ^{13}C NMR (126 MHz, $CDCl_3$): δ 176.4, 169.7, 155.1, 135.1, 128.6, 128.5, 128.3, 127.9, 80.2, 67.5, 66.0, 64.1, 53.2, 34.0, 33.7, 28.3, 19.0, 18.9, 18.8.

N-Boc-O-(*S*)-hexyl-L-serine benzyl ester

^{1}H-NMR ($CDCl_3$, 500 MHz): δ [ppm] = 7.36-7.30 (m, 5H), 5.33 (d, J = 8.5 Hz, 1H), 5.24 (d, J = 12.2 Hz, 1H), 5.17 (d, J = 12.2 Hz, 1H), 4.63 (dt, J = 8.2, 3.8 Hz, 1H), 4.50 (dd, J = 11.2, 4.0, 1H), 4.33 (dd, J = 11.2, 3.5, 1H), 2.22 (q, J = 7.4, 2H), 1.57 (q, J = 7.5 Hz, 2H), 1.47 (s, 9H), 1.36-1.24 (m, 4H), 0.91 (t, J = 7.4 Hz, 3H). ^{13}C NMR (126 MHz, $CDCl_3$): δ 173.3, 169.7, 155.1, 135.1, 128.6, 128.5, 128.3, 80.3, 67.5, 64.1, 53.1, 33.8, 31.2, 28.3, 24.4, 22.3, 13.9.

O-(*S*)-Isobutyryl-L-serine benzyl ester

^{1}H-NMR ($CDCl_3$, 500 MHz): δ [ppm] = 8.02 (br, 2H), 7.37-7.30 (m, 5H), 5.26 (d, J = 12.0 Hz, 1H), 5.19 (d, J = 12.0 Hz, 1H), 4.57 (dd, J = 3.6, 2.2 Hz, 2H), 4.39 (t, J = 3.6, 1H), 2.47 (p, J = 7.0 Hz, 1H), 1.19 (d, J = 7.0 Hz, 1H), 1.05 (dd, J = 9.8, 7.0 Hz, 6H). ^{13}C NMR (126 MHz, $CDCl_3$): δ 173.3, 166.7, 134.0, 129.0, 128.7, 128.6, 69.0, 61.1, 52.8, 35.2, 17.9, 13.3.

O-(S)-Hexyl -L-serine benzyl ester

^{1}H-NMR ($CDCl_3$, 500 MHz): δ [ppm] = 7.37-7.30 (m, 5H), 5.25 (d, J = 12.0 Hz, 1H), 5.19 (d, J = 12.0 Hz, 1H), 4.56 (d, J = 3.6 Hz, 2H), 4.33 (t, J = 3.6, 1H), 2.25-2.12 (m, 2H), 1.51 (p, J = 7.6 Hz, 2H),1.33- 1.18 (m, 4H) 0.87 (t, J = 7.2 Hz, 3H). ^{13}C NMR (126 MHz, $CDCl_3$): δ 173.4, 166.9, 134.1, 128.9, 128.7, 128.5, 68.8, 61.1, 52.7, 33.3, 31.1, 24.1, 22.2, 13.8.

N-4-Methylpentyl-O-(*S*)-isobutyryl-L-serine benzyl ester

^{1}H-NMR ($CDCl_3$, 500 MHz): δ [ppm] =11.04. (br, 1H), 7.40-7.30 (m, 5H), 6.52 (d, J = 7.6 Hz, 1H), 5.24-5.14 (m, 2H), 4.90 (dt, J = 7.6, 3.6 HZ, 1H), 4.49 (dd, J = 11.6, 4.0 Hz, 1H), 4.37 (dd, J = 11.6, 3.4, 1H), 2.45 (dq, J = 14.0, 7.0, 1H), 2.32-2.26 (m, 2H), 1.60-1.48 (m, 3H), 1.08 (dd, J = 7.0, 5.6 Hz, 6H), 0.89 (d, J = 6.4 Hz, 6H). ^{13}C NMR (126 MHz, $CDCl_3$): δ 176.8, 174.6, 169.2, 134.8, 128.7, 128.4, 67.8, 63.5, 52.3, 34.4, 33.8, 27.7, 22.2, 18.8, 18.7.

N-4-Methylpentyl-O-(*S*)-hexyl-L-serine benzyl ester

^{1}H-NMR ($CDCl_3$, 500 MHz): δ [ppm] =7.34-7.25 (m, 5H), 6.42 (d, J = 7.8 Hz, 1H), 5.19-5.08 (m, 2H), 4.87 (dt, J = 7.8, 3.8 HZ, 1H), 4.44 (dd, J = 11.4, 4.0 Hz, 1H), 4.30 (dd, J = 11.4, 3.6, 1H), 2.23-2.18 (m, 2H), 2.15 (td, J = 7.6, 3.0 2H), 1.56-1.45 (m, 5H), 1.30-1.17 (m, 4H) 0.87-0.82 (m, 9H). ^{13}C NMR (126 MHz, $CDCl_3$): δ 173.2, 173.1, 169.5, 135.1, 128.6, 128.5, 128.3, 67.5, 63.7, 51.7, 34.3, 34.3, 33.8, 31.2, 27.7, 24.4, 22.3, 22.2, 22.2, 13.9.

N-4-Methylpentyl-O-(*S*)-isobutyryl-L-serine

^{1}H-NMR ($CDCl_3$, 500 MHz): δ [ppm] =10.20. (br, 1H), 6.56 (d, J = 7.0 Hz, 1H), 4.86 (q, J = 3.6 HZ, 1H), 4.47 (qd, J = 11.6, 3.4, 2H), 2.58 (dq, J = 14.0, 7.0, 1H), 2.32-2.26 (m, 2H), 1.60-1.48 (m, 3H), 1.17 (dd, J = 7.0, 2.6 Hz, 6H), 0.92 (d, J = 6.4 Hz, 6H). ^{13}C NMR (101 MHz, $CDCl_3$): δ 177.1, 174.7, 171.8, 63.5, 52.2, 34.4, 34.3, 33.9, 27.7, 22.2, 22.2, 18.9.

N-4-Methylpentyl-O-(*S*)-hexyl-L-serine

^{1}H-NMR ($CDCl_3$, 500 MHz): δ [ppm] = 9.83 (br, 1H),6.49 (d, J = 7.4 Hz, 1H), 4.84 (m, 1H), 4.49 (dd, J = 11.6, 4.2 Hz, 1H), 4.42 (dd, J = 11.6, 3.4, 1H), 2.29 (dt, J = 18.8, 7.6 Hz, 4H), 1.57 (ddt, J = 37.6, 14.8, 7.4 Hz, 5H), 1.29 (tt, J = 8.4, 5.4 Hz, 4H), 0.89 (dd, J = 10.0, 6.4 Hz, 9H). ^{13}C NMR (126 MHz, CDCl3) δ 174.6, 173.7, 171.6, 63.6, 51.9, 34.3, 33.9, 31.2, 27.7, 24.4, 22.2, 22.2, 22.1, 13.8.

6-Methylheptanamide

6-Methylheptanoic acid (1 mmol) in dry DCM (3 mL), followed by dropwise addition of $SOCl_2$ (1.3 mmol, 0.094 mL), was added to a single-neck, round-bottom flask (10 mL) fitted with a reflux condenser and a calcium chloride-filled-guard tube. The reaction mixture was kept 6 h at 60 ºC and was then subjected to rotary evaporation under reduced pressure to remove solvent and excess $SOCl_2$, affording 6-methylheptanoyl chloride. The product was directly used in the next reaction step without further purification. The crude acid chloride was dissolved in THF and the mixture was added dropwise to aq NH_3 (14.8M, 2 mL) at 0 ºC. The mixture was allowed to warm to room temperature (rt), stirred overnight, and then diluted with DCM. The organic and aqueous layers were separated, and the aqueous layer was extracted twice with DCM. The combined organic layers were washed with brine, dried over Na_2SO_4, and filtered. The solvent was removed under reduced pressure to afford the crude amide which was purified via flash column

chromatography on silica gel to yield 6-methylheptanamide (110 mg, 77%). ^{1}H NMR (500 MHz, $CDCl_3$) δ 5.46 (s, 1H), 2.24 (t, *J* = 7.6 Hz, 2H), 1.63-1.57 (3H, m), 1.33 –1.24 (4H, m), 0.87 (d, *J* = 6.6 Hz, 6H).

Octanamide

Starting with octanoic acid and following the procedure described for 6-methylheptanamide, octanamide was obtained (113 mg, 79% yield). ^{1}H NMR (500 MHz, $CDCl_3$) δ 5.47 (s, 2H), 2.24 (t, *J* = 7.6 Hz, 2H), 1.63 (h, *J* = 7.2 Hz, 2H), 1.36 – 1.24 (m, 8H), 0.92 – 0.83 (m, 3H).

4,6-Dimethylheptanamide

To a solution of 6-methyl-4-methyleneheptanoic acid (0.78 g, 5.0 mmol) in anhydrous MeOH (50 mL), 10% Pd/C (250 mg) was added in one portion. The black slurry was stirred under H_2 atmosphere (balloon) overnight before being filtered through celite. The filtrate was concentrated to give 4,6-dimethylheptanoic acid as a light yellow oil (Chen et al., 2017).

Starting with 4,6-dimethylheptanoic acid and following the procedure described for 6-methylheptanamide, 4,6-dimethylheptanamide was obtained (108 mg, 69% yield). ^{1}H NMR (500 MHz, $CDCl_3$) δ 2.30 -2.18 (m, 2H), 1.70 - 1.62 (m, 2H), 1.58-1.49 (m, 1H), 1.48 - 1.40 (m, 1H), 1.15 - 1.09(m, 1H), 1.06 - 0.97 (m, 1H), 0.87 (dd, *J* = 6.6, 1.5 Hz, 6H), 0.84 (d, *J* = 6.6 Hz, 3H).

Methyl (4-methylpentanoyl)serinate

4-Methylpentanoic acid (Sigma-Aldrich) (1 mmol, 1 eq., 116 mg) was added to a stirred mixture of N-methyl-serinate (Sigma-Aldrich) (1 mmol, 1 eq., 119 mg) in dichloromethane (10 mL). N,N'-dicyclohexylcarbodiimide (1 mmol, 1 eq., 206 mg) was added followed by triethylamine (1 mmol, 1 eq., 101 mg). The reaction mixture was stirred 12 h at ambient temperature. The reaction product was purified by column chromatography with pentane/diethyl ether (2/1) as eluent to give methyl (4-methylpentanoyl)serinate (173 mg, 80 %).

(4-Methylpentanoyl)serine (= *N*-4-Methylvaleroyl-L-serine)

To a solution of methyl (4-methylpentanoyl)serinate (173 mg, 0.8 mmol) in MeOH (3 mL) was added a solution of LiOH (96 mg, 4 mmol) in H_2O (1 mL) at 0 °C. After stirring 2 h at ambient temperature, the reaction was quenched with concentrated aqueous HCl. The mixture was extracted with AcOEt, and the organic layers were dried over anhydrous Na_2SO_4, filtered and

concentrated. The residue was purified by column chromatography on silica gel with hexane/EtOAc (4/1) as eluent to give (4-methylpentanoyl)serine (146 mg, 90% yield).

Reference

Chen D-F, Chu JCK, and Rovis T (2017) Directed γ-C(sp^3)–H alkylation of carboxylic acid derivatives through visible light photoredox catalysis. *Journal of the American Chemical Society 139* (42), 14897-14900. DOI: 10.1021/jacs.7b09306
