# Supplementary material for: Origin, structure and functional transition of sex pheromone components in a false widow spider
Source: Commun Biol. 2022 Oct 30;5:1156. doi: 10.1038/s42003-022-04072-7 (PMC9618557; doi:10.1038/s42003-022-04072-7)
Supplement: Supplementary file 3 — Description of Additional Supplementary Files [file 42003_2022_4072_MOESM3_ESM.pdf]

Description of Additional Supplementary Files

File name: Supplementary Data 1
Description: All data, including raw data, underlying the figures.
